# Climate Regimes, El Niño-Southern Oscillation, and Meningococcal Meningitis Epidemics

**DOI:** 10.3389/fpubh.2015.00187

**Published:** 2015-07-30

**Authors:** Olusegun Steven Ayodele Oluwole

**Affiliations:** ^1^Neurology Unit, College of Medicine, University of Ibadan, Ibadan, Nigeria

**Keywords:** El Niño, meningococcal, meningitis, harmattan, climate, Sahel, pacific decadal oscillation

## Abstract

Meningococcal meningitis is a major public health problem that kills thousands annually in Africa, Europe, North, and South America. Occurrence is, however, highest during the dry seasons in Sahel Africa. Interannual changes in precipitation correlate with interannual changes in El Niño-Southern Oscillation (ENSO), while interdecadal changes in precipitation correlate with Pacific Decadal Oscillation (PDO). The objective of the study was to determine if there is spectral coherence of seasonal, interannual, and interdecadal changes in occurrence of meningococcal meningitis in Sahel, Central, and East Africa with interannual and interdecadal changes of PDO and ENSO. Time series were fitted to occurrence of meningococcal meningitis in Sahel, Central, and East Africa, to indices of precipitation anomalies in the Sahel, and to indices of ENSO and PDO anomalies. Morlet wavelet was used to transform the time series to frequency-time domain. Wavelet spectra and coherence analyses were performed. Occurrence of meningococcal meningitis showed seasonal, interannual, and interdecadal changes. The magnitude of occurrence was higher during warm climate regime, and strong El Niños. Spectra coherence of interannual and interdecadal changes of ENSO and PDO with occurrence of meningococcal meningitis in Sahel, Central, and East Africa were significant at *p* < 0.0001. Precipitation in Sahel was low during warm climate regimes. Spectra coherence of changes in precipitation in Sahel with ENSO was significant at *p* < 0.0001. ENSO and PDO are determinants of the seasonal, interannual, and interdecadal changes in occurrence of meningococcal meningitis. Public health management of epidemics of meningococcal meningitis should include forecast models of changes in ENSO to predict periods of low precipitation, which initiate occurrence.

## Introduction

Meningococcal meningitis is a bacterial meningitis caused by *Neisseria meningitidis*, which is non-spore-forming, non-motile, gram-negative cocci. While headache, fever, nausea, and vomiting occur in meningococcal meningitis as in other febrile illnesses, shock, petechiae and purpuric skin rashes are its major characteristics (1, 2). Case fatality may exceed 20% (1), and severe neurological sequelae exceeded 5% (3) in a resource-rich setting. Seasonal epidemics of meningococcal meningitis occur worldwide (4), but its burden of disease in Africa is estimated to exceed that of trypanosomiasis, Chagas disease, schistosomiasis, leishmaniasis, lymphatic filariasis, onchocerciasis, leprosy, and dengue combined (5). Historically, meningococcal meningitis was first described in Europe in 1805, in USA in 1806, and in Sahel Africa in 1905, but several epidemics and pandemics occurred in the 19th and 20th centuries (6). In 1963, the meningitis belt was described in Sahel Africa (7), where most cases occur annually during the dry seasons. In addition to seasonal epidemics, cyclical epidemics occur every 8–12 in the Sahel (1), but the determinants are unknown.

Intensities of El Niño, the warm phase of El Niño-Southern Oscillation (ENSO), determine changes in precipitation in the Sahel (8), several parts of Africa (9), and other parts of the world (10). The intensities of ENSO vary on interannual and interdecadal timescales, while the Pacific Decadal Oscillation (PDO), which modulates the ENSO, varies predominantly on interdecadal timescale (11). Climate regimes are defined by the warm or cool modes of the PDO, which persists for decades (12). El Niños dominate warm climate regimes, while La Niñas, the cool phase of ENSO, dominate cool climate regimes (13). The strong relationship of low precipitation to occurrence of meningococcal meningitis, which is highest in the arid to semi-arid Sahel where annual rainfall is between 100 mm and 600 mm (14), suggests that determinants of global changes in precipitation will covary with the epidemics. The objective of the study was to determine if there is spectra coherence of seasonal, interannual, and interdecadal changes in occurrence of meningococcal meningitis in Sahel, Central, and East Africa with interannual and interdecadal changes in PDO and ENSO.

## Materials and Methods

### Study sites

Countries in the Sahel are Senegal, Gambia, Guinea, Mauritania, Mali, Benin, Burkina Fasso, Nigeria, Cameroon, Niger, Tchad, Sudan, and Eritrea; countries in Central Africa are Burundi, Central African Republic, Rwanda, Angola, Congo, Democratic Republic of the Congo, Sao Tome and Principe, Equatorial Guinea, and Gabon; while countries in East Africa are Tanzania, Uganda, Ethiopia, Djibouti, Kenya, Somalia, Comoros, Malawi, Zambia, Zimbabwe, Seychelles, Mozambique, Madagascar, and Mauritius.

### Indices of PDO, ENSO, and Sahel precipitation anomalies

Multivariate El Niño-Southern Oscillation Index (MEI) data, which were computed from sea-level pressure, zonal and meridional components of the surface wind, sea surface temperature, surface air temperature, and total cloudiness fraction of the sky of the South Pacific Ocean (15) from 1950 to 2014 were downloaded from the website of National Oceanic and Atmospheric Administration^1^ (NOAA), USA, while intensities of El Niños from 1950 to present time were assessed using the Oceanic Niño Index (ONI) downloaded from http://ggweather.com/enso/oni.htm. Ranks of El Niño from 1871 to 1949 from http://www.esrl.noaa.gov/psd/enso/mei.ext/rank.ext.html, and from 1950 to 2014 from http://www.esrl.noaa.gov/psd/enso/mei/table.html. Pacific Decadal Oscillation Index was downloaded from the Joint Institute for the Study of the Atmosphere and Ocean Arctic Oscillation website^2^.

Sahel precipitation data from 1900 to 2013 were obtained from the Joint Institute for the Study of the Atmosphere and Ocean (16). The precipitation index is anomalies with respect to 1950–79.

### Meningococcal meningitis epidemics data

Monthly data of occurrence of meningococcal meningitis cases in the Sahel, from 2006 to 2014, were obtained from World Health Organisation Weekly Meningococcal Meningitis Surveillance Bulletins^3^. Annual data of occurrence of meningococcal meningitis, from 1965 to 2010, in the Sahel, Central, and East African regions were obtained from World Health Organisation Global Health Observatory Data Repository^4^.

### Time series of climate indices and occurrence data

Time series were fitted to indices of ENSO, PDO, and Sahel precipitation, and to occurrence of meningitis. Autocorrelation and partial autocorrelation tests, and lag plots were applied to exclude white noise and to inspect for seasonality. Stationarity was assessed using the unit root test. To determine month of peak occurrence of meningitis annually, the monthly data from 2006 to 2014 were rescaled to 0–1 values for each year, and plotted.

### Time-frequency domain analysis

Wavelet methods which have been applied to epidemiological (17, 18), human (19), and environmental data (20, 21) were applied to the time series of this study. Time series were transformed to time-frequency domain using Morlet wavelet, which was defined as follows (17, 18):

$$\psi_{0}\left(\eta \right)=\pi^{-1/4}e^{i\omega_{0}\eta }e^{-\eta^{2}/2}$$

where *ω*_0_ is dimensionless frequency, and *η* is dimensionless time, was used to transform the time series. The continuous wavelet transform of time series (*x_n_*, *n* = 1,…, *N*)

Wnx(s)=δts∑n′=1N xn′ψ0n′−nδts

with uniform time steps *δt* was defined as the convolution of *x_n_* with the scaled and normalized wavelet (17, 18). The cross wavelet transform was defined as follows (17, 18):

$$D\left(\frac{\left|W_{n}^{x}\left(s\right)W_{n}^{y}⋆\left(s\right)\right|}{\sigma_{x}\sigma_{y}}<p\right)=\frac{Z_{v}\left(p\right)}{v}\sqrt{P_{k}^{x}P_{k}^{y}}$$

where *Z_v_* (*p*) was the confidence level associated with probability *p*, and $P_{k}^{x}$ and $P_{k}^{y}$ were the power spectra. The wavelet coherency phase was (17, 18)

$$R_{n}^{2}\left(s\right)=\frac{|S\left(s^{-1}W_{n}^{\mathrm{xy}}\left(s\right)\right){|}^{2}}{S\left(s^{-1}|W_{n}^{x}\left(s\right){|}^{2}\right)\cdot S\left(s^{-1}|W_{n}^{y}\left(s\right){|}^{2}\right)}$$

The global wavelet spectra, the equivalent of the Fourier power spectrum smoothed by the Morlet wavelet function (17, 22), were

$${\bar{W}}^{2}\left(s\right)=\frac{1}{N}\sum_{n=0}^{N-1}{\left|W_{n}\left(s\right)\right|}^{2}$$

Phase plots were drawn to determine phase shift between the time series.

### Granger causality test

The Granger causality test was performed to determine if ENSO precedes occurrence of meningococcal, or otherwise. The model used for the calculation is as follows:

$y_{i,t}=\alpha +\sum_{l=1}^{p}\beta_{l}y_{i,t-12}+\gamma_{l}x_{i,t-12}+\varepsilon_{i,t}$. The data used were the monthly occurrence of meningococcal meningitis in the Sahel.

### Historical meningococcal epidemics El Niños

Historical occurrence data of meningococcal meningitis before 1965, the commencement of this study, were obtained from the literature (6, 23). The strength of El Niño at the time of each epidemic was determined.

### WHO criteria for associating ENSO with health

The following criteria of the WHO for linking health problems with ENSO activity (24) were considered in the design of this study: (a) Climatological evidence of appropriate teleconnections or direct effects of ENSO in the area of interest. (b) Biological evidence that the diseases or other health impacts of interest have a plausible biological link with weather exposures (precipitation, land surface temperature, sea surface temperature), e.g., field studies of vectors which examine their abundance in relation to the ENSO cycle. (c) Statistical analyses which show that disease incidence or epidemics vary over time with the ENSO cycle.

### Statistics

Statistical analyses were performed using the R Statistical Programing and Environment (25). Specifically, wavelet analyses were performed using the biwavelet package, phase plots using the WaveletComp package, and graphics using the ggplot2 package. Statistical significance of spectra periodicity was determined as defined in (17, 18). Briefly the time series is assumed to have a mean power spectrum, which is possibly red noise. The null hypothesis is that any peak on the power spectrum is not significant above the background spectrum. The desired level of significance in this study was set at 0.01% level. The R MSBVAR package was used for Granger causality test.

## Results

### Monthly meningococcal meningitis cases in Sahel

**Figure 1:**
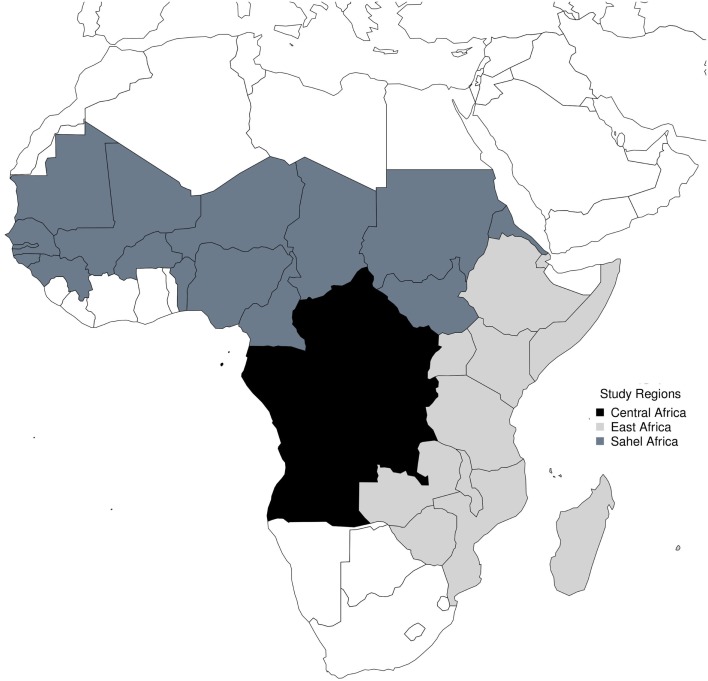
**Map of study sites**.

**Figure 2:**
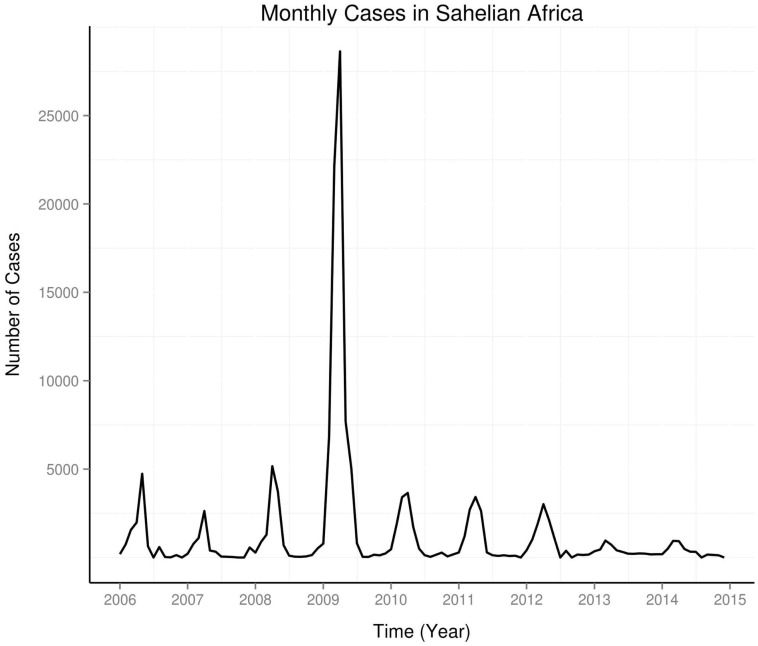
**Time series of monthly occurrence in Sahel**.

**Figure 3:**
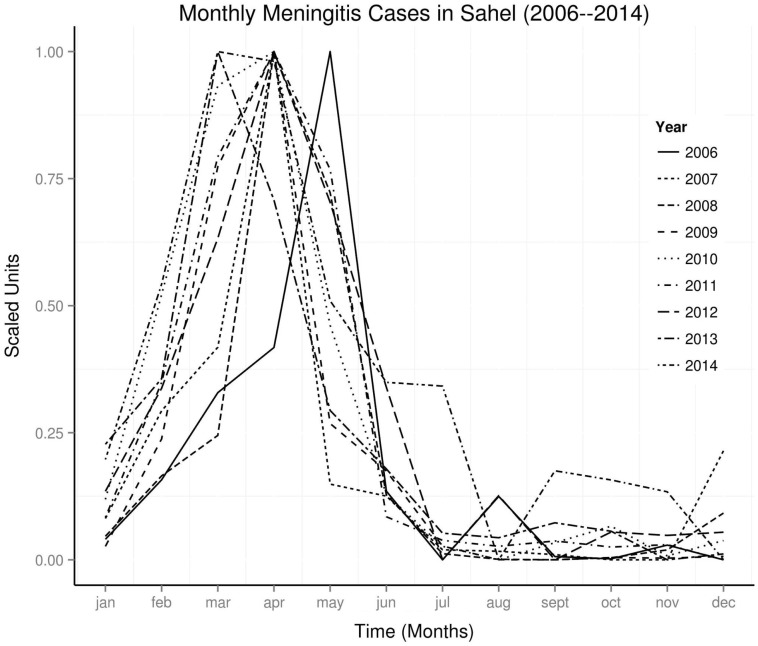
**Seasonal peaks of occurrence in Sahel**.

The study areas are shown in Figure 1. Time series of monthly occurrence data in the Sahel shows regular annual peaks, but a very large peak in 2009 (Figure 2). Amplitude of peaks reduced from 2012 to 2014. Yearly plots of the monthly data show highest peaks of occurrence from March to May, and very low peaks from August to November (Figure 3). El Niños during the study period were strongest in 2009–2010, while La Niñas dominated the period of lowest amplitude of occurrence from 2012 to 2014.

**Figure 4:**
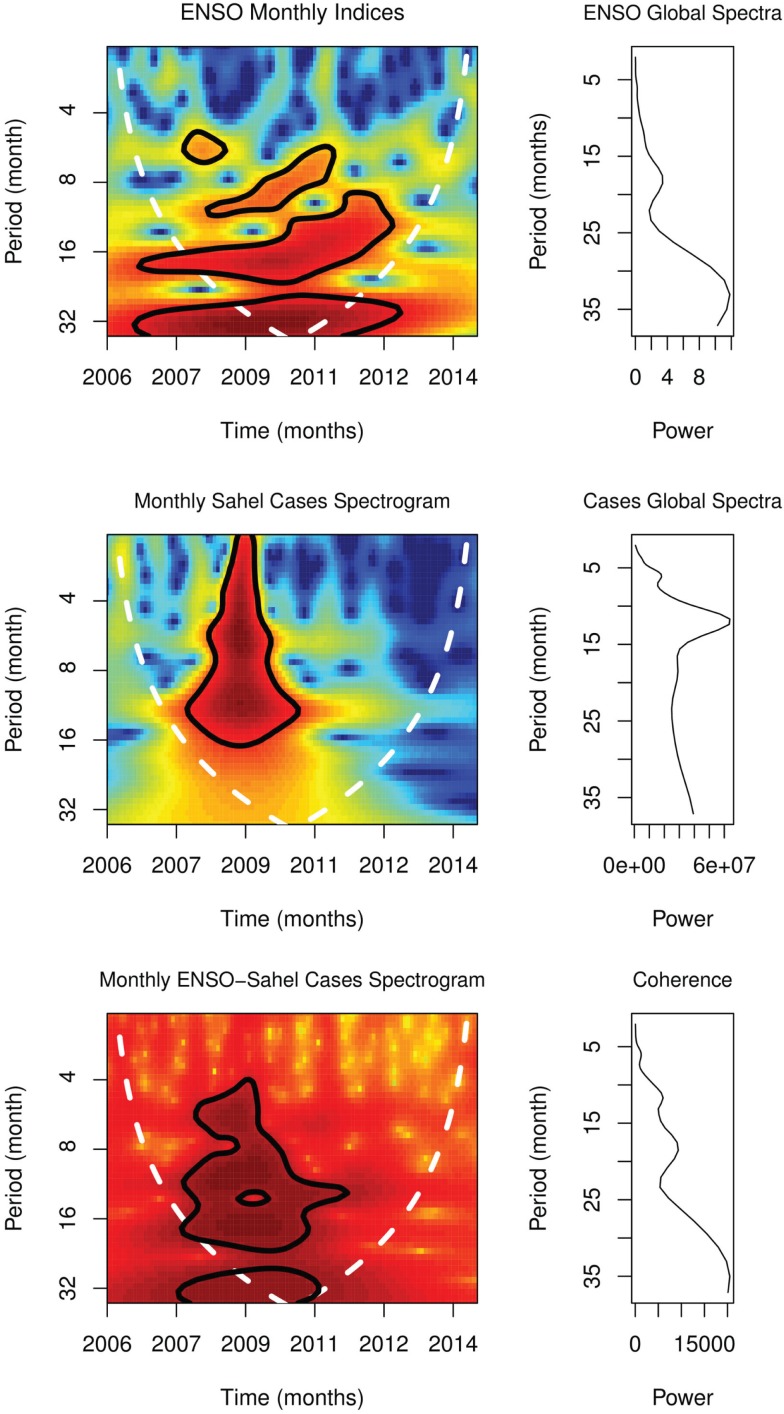
**Spectra of occurrence in Sahel**.

Global spectra of monthly cases showed peaks at 6 and 12 months, but at 18 and 36 months for monthly ENSO anomalies. Spectrogram of monthly cases showed significant periodicity at 12 months from September 2007 to May 2009 and at 6 months from July 2008 to March 2010, *p* < 0.0001, while spectrogram of ENSO showed significant periodicity at 6 months from December 2008 to October 2009, at 12 months from July 2008 to January 2013, and at 24 from November 2006 to September 2012, *p* < 0.0001 (Figure 4). Peaks of coherence squared were at 6, 12, 18, and 34 months (Figure 4). Cross wavelet spectrogram of monthly cases and monthly ENSO indices showed significant periodicity at 6, 12, and 18 months from 2007 to 2011, *p* < 0.0001.

### Climate regime and occurrence

**Figure 5:**
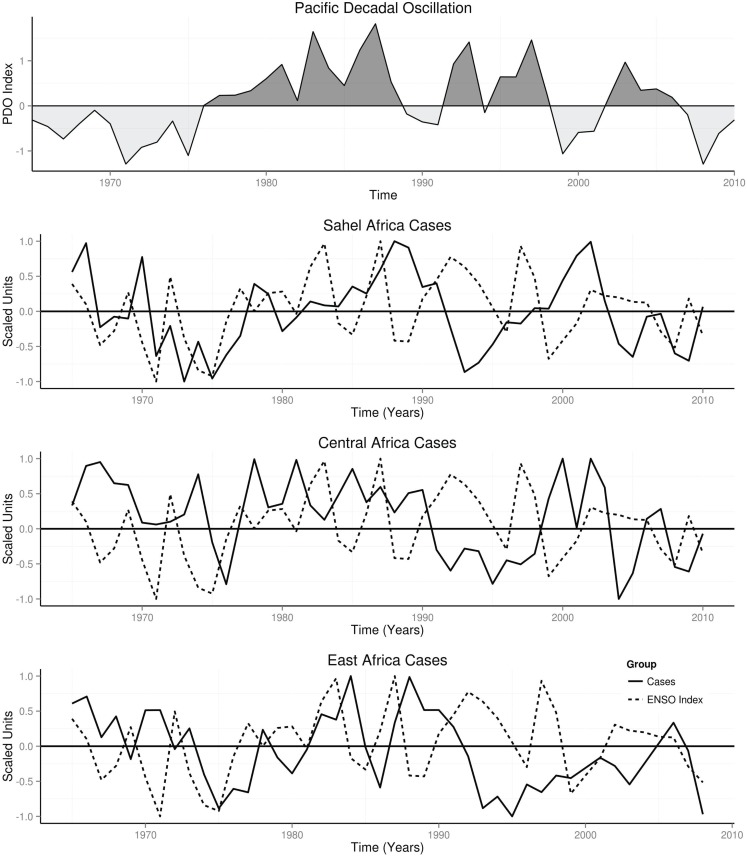
**Annual time series of occurrence, PDO, and ENSO**.

PDO was in warm mode from 1976 to 2006, but dipped to cool mode briefly in 1995 (Figure 5). Annual cases declined during the cool mode, but were above the mean during the warm mode in the three study areas (Figure 5). Annual cases were above the mean in Sahel from 1978 to 2003 with a dip in 1990, in Central from 1975 to 2004 with a dip in 1995, and in East Africa from 1976 to 2004 with a dip in 1995 (Figure 5).

**Figure 6:**
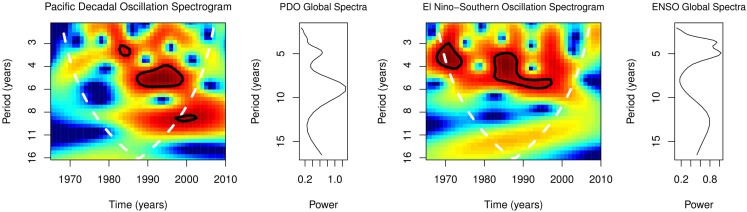
**Spectral of PDO and ENSO**.

**Figure 7:**
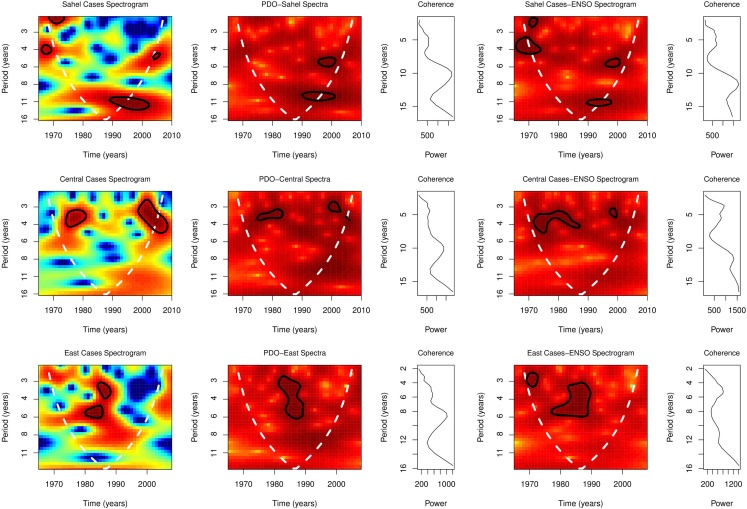
**Spectral of annual occurrence, PDO, and ENSO**.

Global spectra of PDO from 1965 to 2010 showed peaks at 5.0–9.0 years (Figure 6). Spectrograms showed statistically significant time-varying periodicities of 3–11 years for PDO, *p* < 0.0001 (Figure 6). Coherence squared of PDO and cases were at 5.0 and 10 years in the Sahel, 4.0 and 10.0 years in Central Africa, and 3.0, 6.0, and 9.0 years in East Africa (Figure 7). Cross spectrogram of occurrence and PDO showed significant coherence at time-varying periodicities of 3–11 years, *p* < 0.0001 (Figure 7). Details of the periodicities are shown in Table 1.

**Table 1:** **Significant Periodicities of Cases and Climate Indices**.

Spectrograms			
	Period^a^ 1 (time)	Period 2 (time)	Period 3 (time)
**Climate indices**			
ENSO^b^	3.0–4.0 (1970–1975)	3.5–5.0 (1982–1988)	5.0–6.0 (1982–1998)
PDO^c^	3.0–4.0 (1982–1985)	4.0–6.0 (1985–2000)	8.0–10.0 (1995)
**Cases**			
Sahel	1.0–2.0 (1972–1975)	10.0–12.0 (1988–1991)	5.0 (2002)
Central	3.0–4.0 (1975–1982)	3.0–3.5 (1995–1998)	3.5–5.0 (1995–2005)
East	5.0–6.5 (1978–1986)	3.0–4.0 (1985–1988)	4.0–5.0 (1986–1988)
**Cases-ENSO^d^**			
Sahel	1.0–4.5 (1972–1975)	10.5–11.5 (1988–1995)	5.0–6.0 (1995–2000)
Central	3.5–5.0 (1975–1978)	3.5–4.5 (1980–1988)	3.0–4.0 (1995–2000)
East	2.0–3.5 (1972–1975)	4.0–6.0 (1978–1985)	3.0–6.0 (1985–1988)
**Cases-PDO^e^**			
Sahel	10.0–11.0 (1988–1995)	5.0–6.5 (1993–1995)	5.0–7.0 (1995–2002)
Central	3.5–4.0 (1975–1985)	5.0 (1988–1992)	3.0–4.5 (1995–2005)
East	3.0–4.0 (1982–1988)	4.0–7.0 (1984–1990)	9.0 (1992–1994)

*^a^Periodicities in years*.

*^b^El Niño-Southern Oscillation*.

*^c^Pacific Decadal Oscillation*.

*^d^Cross wavelet spectra of cases and Indices of El Nino-Southern Oscillation*.

*^e^Cross wavelet spectra of cases and Indices of Pacific Decadal Oscillation*.

### ENSO and occurrence

Time series plots of annual cases and ENSO indices in Sahel, Central, and East Africa showed periodic changes, but the time series of cases are phase shifted to the right (Figure 5). Global spectra of ENSO from 1965 to 2010 showed peaks at 4.0, 5.0, and 12.0 years (Figure 6), while its spectrogram showed significant time-varying periodicities of 3–6 years, *p* < 0.0001.

Global spectra of annual cases showed peaks at 4.0, 6.0, and 11.0 years in the Sahel, 3.7, 6.0, and 12.0 years in Central, and 3.0, 6.0, and 9.0 years in East Africa (Figure 7). Spectrograms showed statistically significant time-varying periodicities of 1–12 years for occurrence in Sahel, Central, and East Africa, *p* < 0.0001, (Figure 7).

Global spectra of coherence of ENSO and cases were at 4.5, 6.0, and 12.0 years in the Sahel, 3.7, 6.0, and 12 years in Central Africa, and 5.0 and 11.0 years in East Africa (Figure 7). Cross spectrogram of occurrence and ENSO showed significant coherence at time-varying periodicities of 1–12 years, *p* < 0.0001, (Figure 7). Details of the periodicities are shown in Table 1.

Granger causality test showed that ENSO Granger caused meningococcal meningitis series (*p* < 0.001), but meningococcal meningitis did not Granger cause ENSO series (*p* > 0.05).

### Historical meningococcal epidemics and El Niños

Pandemics or major epidemics of meningococcal meningitis occurred in 1905 in West Africa (26), in the USA in 1918–1919 and 1930–1931 (27, 28), in England in 1915 and 1931 (29), and in 1941 in Africa, Europe, and North America (30). Of a range of ranks from 1 to 135, the median ranks (50% IQR) of ENSO index from August 1905 to May 1906 was 117 (99–124), from August 1918 to May 1919 was 118 (116–125), and from August 1930 to May 1931 was 130 (127–132).

### Precipitation changes in the Sahel

**Figure 8:**
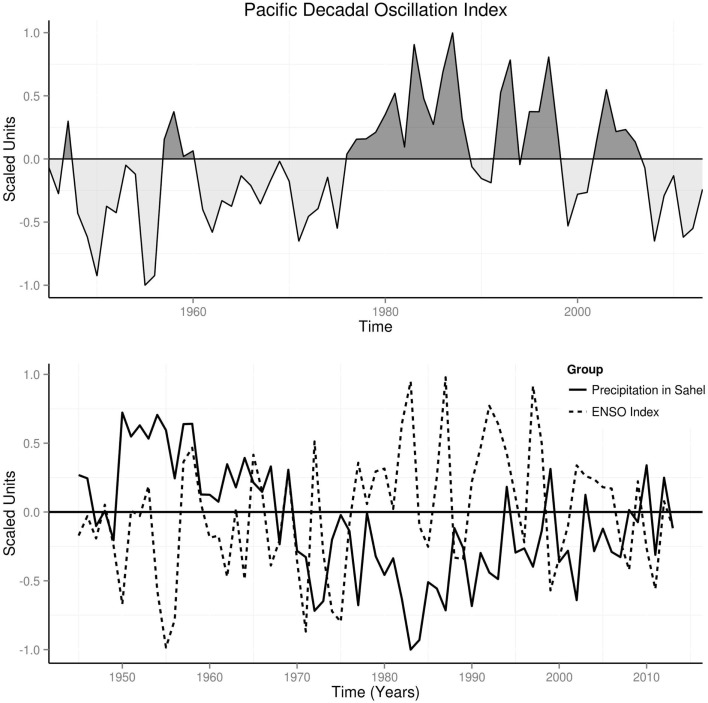
**Annual time series of precipitation in the Sahel, PDO, and ENSO**.

Precipitation was above the mean from 1950 to 1970, during cool climate regime, but below the mean from 1972 to 1998 during warm climate regime (Figure 8). La Niñas dominated the period of high precipitation, while El Niños dominated the period of low precipitation (Figure 8). Precipitation returned toward mean from 2000 to 2013 since the amplitudes of El Niños have reduced, and replaced by more La Niñas (Figure 8).

**Figure 9:**
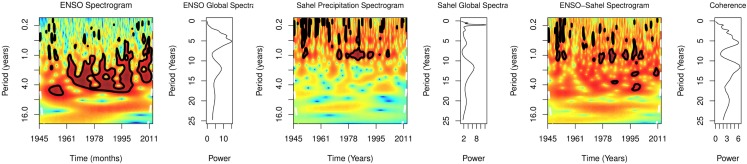
**Spectral of precipitation in the Sahel and ENSO**.

Global spectra from 1945 to 2013 showed peaks at 1.0, 3.0, 6.0, 12.0, and 20 years for precipitation changes in Sahel, but at 3.0, 6.0, 12.0, and 20.0 years for ENSO (Figure 9). Spectrograms showed statistically significant time-varying periodicities of 1–6 years for ENSO, and 1 year for Sahel precipitation, *p* < 0.0001 (Figure 9). Peaks of coherence squared were at 1.0, 3.0, 6.0, 12.0, and 20.0 years. Cross spectrogram showed significant time-varying coherence at 1–6 years, *p* < 0.0001 (Figure 9).

## Discussion

This study shows periodic changes in occurrence of meningococcal meningitis and indices of ENSO and PDO. Periodic changes in occurrence of meningococcal meningitis have been observed in the USA (31), in United Kingdom (29), and in Sahelian countries (7); Chad (32), Mali (33), and Burkina Faso (34). Seasonal epidemics of meningococcal meningitis in the Sahel is known to peak from February to May annually (35). In this study, seasonal epidemics of meningococcal meningitis in the Sahel between 2006 and 2014 peak from March to May, but April peak is the commonest (Figure 3). Although occurrence fell almost to none outside of the peak period, there are low amplitude peaks from August to November, which indicate biannual occurrence (Figure 3). The periods of peak occurrence coincide with periods of seasonally low precipitation. Although peaks of varying amplitudes occurred annually from 2006 to 2014, a very large peak, which occurred in 2009, illustrates the appearance of unusually large peaks of occurrence at unpredictable periods, which have been observed since the 1930s (36) (Figure 2). Occurrence of this large peak, however, coincided with the high intensity El Niño of 2009–2010. Furthermore, the period of very low peaks, from 2013 to 2014, also coincided with the period of La Niña activity from 2012 to 2014, when no El Niño occurred. Thus, the monthly data from Sahel show strong relationship of seasonality of epidemics of meningococcal meningitis and ENSO activity.

The spectra of monthly time-series in Sahel, which show similar time-varying periodicities of 6 and 12 months with the spectra of ENSO, indicate that both variables share rhythms at similar timescales (Figure 4). Strong spectra coherence indicates that the two rhythms are strongly correlated (Figure 4). The ENSO Granger causality, which shows that changes in ENSO precede occurrence, indicate that the relationship of ENSO and occurrence is not bidirectional. Since variability of ENSO is linked to changes in precipitation (10), coherence of seasonal changes in occurrence and ENSO indicate that precipitation changes induced by ENSO are the underlying determinant of the epidemics.

### Climate regimes and epidemics of meningococcal meningitis

In the past century cool climate regimes occurred from 1890 to 1924 and from 1947 to 1976, while warm climate regime occurred from 1925 to 1946, and from 1977 to 1998 (12). Time series of occurrence of meningococcal meningitis in the USA from 1913 to 1931 showed two peaks from 1913 to 1924 during cool climate regime, but five peaks, which increased steadily in amplitude from 1925 to 1931, during warm climate regime (36). This showed that higher number of epidemics occurred in 7 years of warm climate regime compared with 12 years of cool climate regime. In this study, decline of occurrence during cool climate regime and increase above the mean during warm climate regime in the Sahel, Central, and East Africa, indicates that strong association of climate regime and the epidemics exists (Figure 5). Thus, warm climate regime favors occurrence of meningococcal meningitis epidemics.

Wavelet analysis, which transforms time series to time-frequency domain, has shown the time-varying nature of PDO (37). During the period of this study, from 1965 to 2010, the dominant periodicities of 5 and 9 years for PDO, shows its relatively long period of variability (Figure 6). Spectrogram, however, shows significant time-varying periodicities of 3–11 years (Figure 6). Significant spectral coherence of PDO and occurrence, periodicities of 3–11 years, indicates that climate regime and occurrence are coupled (Figure 7).

### ENSO and epidemics of meningococcal meningitis

Cycles of occurrence of meningococcal meningitis in Sahel, Central, and East Africa show that El Niños were accompanied by high peaks of occurrence, while La Niñas were accompanied by low peaks of occurrence. Furthermore, phases of occurrence are right shifted relative to that of ENSO (Figure 5). Thus, peaks and troughs of occurrence follow variations of the ENSO.

Periodicity of the ENSO, like the PDO, is time-varying. Periodicity of ENSO, which was 3–4 years from 1872 to 1910, was 5–7 years from 1911 to 1960, but 5 years from 1970 to 1992 (38). The periodicities of 4–13 years for the ENSO, which is in the same range as that for occurrence 4–12 years, indicate similar rhythms of both variables (Figure 6). In Burkina Faso, Chad, Mali, and Sudan, inter-epidemic periodicity varies from 8 to 12 years from the 1940s to mid-1990s (1). Spectral coherence of ENSO and occurrence indicates that the observed varying inter-epidemic duration is attributable to changes in the periodicities of ENSO (Figure 7). Since interannual changes in precipitation covary with ENSO activity (39), changes in precipitation are the underlying mechanism of the spectra coherence of ENSO and occurrence.

Historical data showed that pandemics or major epidemics of meningococcal meningitis occurred in 1905 in Nigeria (26), in 1918 and 1929–1931 in the USA (27, 28), in 1915–1917 and 1931 in England (29), and in 1941–1942 in Africa, Europe, and North America (30). All these epidemics occurred following strong or moderately strong El Niños, which have been linked to health outcomes (24). Thus, historical and present data indicate strong correlation of ENSO activity and epidemics of meningococcal meningitis.

### Risk factors for epidemics of meningococcal meningitis

Meningococci are commensals of the nasopharynx, but minority of strains cause invasive infection (40). The risk factors for meningococcal meningitis include virulence of the organism, carriage (41), host immunity, overcrowding, and climate (1). Serogroups that cause invasive disease, however, differ in different regions of the world, and change over time (5). Low absolute humidity and dust enhance invasive disease by damaging the mucosal barrier directly or by inhibiting mucosal immune defenses, while travel and migration facilitate the transmission of virulent strains (1). Concurrent upper respiratory tract infections also contribute to invasive disease. While there are several risk factors, serogroups, carriage, and overcrowding do not always predict epidemics (41). Thus, multiple risk factors are necessary for the epidemics to occur.

Cyclicity of meningococcal meningitis epidemics was correlated with sunspots cycles in Bulgaria in 2000 (42) and in the USA in 1946 (43). Sunspots are magnetic storms on the Sun, which appear as dark spots (44). The number of sunspots occurs in cycles, which reach maximum approximately every 11 years (44). Since solar irradiance, which impacts on precipitation (45), correlates with the number of sunspots, the number of sunspots as risk factor for epidemics of meningococcal meningitis can be attributed to changes in precipitation.

ENSO activity has been correlated with the monsoons (39). Since 1970 El Niño has been correlated with droughts and La Niña with increase precipitation in the Sahel (46). In this study reduction of precipitation during warm climate regime, when El Niños were more frequent, shows the strong effects of ENSO on Sahel monsoon in the past 45 years (Figure 8). Thus, the striking increase in precipitation during the cool climate regime from 1945 to 1970 and from 2008 to 2013, and during reduction of the amplitude of El Niños and frequent La Niñas indicate coupling of ENSO activity and changes in precipitation in the Sahel (Figure 8). Periodicities of 6, 12, and 20 years for changes in precipitation in Sahel, agree with documented periods of occurrence of major epidemics in the Sahel (1) (Figure 9). The time-varying nature of the periodicities is shown in the spectrograms, where significant changes occur more frequently during warm than cool climate regime. Although teleconnection of the ENSO in the Sahel has been doubted or considered weak (47), spectral coherence of ENSO and precipitation in the Sahel show otherwise (Figure 9). This finding indicates that changes in precipitation in the Sahel are attributable to ENSO activity.

## Limitations of Study

Since virulence of organism, innate immunity, carriage, overcrowding, and other risk factors were not controlled for in this study, the peaks of occurrence in the time series cannot be attributed to climate alone. Immunization, for example, is expected to contribute to reduction of number of potential cases. Although there are many potential confounding factors, the WHO has documented that these confounding factors are unlikely to consistently covary with ENSO in long time-series (24). Thus, the correlations found in this study are unlikely to be biased by confounding factors.

## Conclusion

Wavelet methods have been used to correlate ENSO activity with periodic epidemics of malaria in West Africa (48), cholera in Bangladesh (49) and West Africa (50), and dengue in Puerto Rico, Mexico, and Thailand (51). In conclusion, this study shows that occurrence of meningococcal meningitis shows seasonal, interannual, and interdecadal variations which covary with climate regimes and ENSO activity. Although forecast models (52) have not consistently predicted occurrence and strength of El Niños, they should become part of public health strategies to anticipate periods of occurrence of high intensities El Niños when major epidemics are likely to occur.

## Conflict of Interest Statement

The author declares that the research was conducted in the absence of any commercial or financial relationships that could be construed as a potential conflict of interest.

## Footnotes

^1^http://www.esrl.noaa.gov/psd/enso/mei/table.html

^2^http://jisao.washington.edu/pdo/PDO.latest

^3^http://www.meningvax.org/epidemic-updates.php

^4^http://apps.who.int/gho/data/node.main.179?lang=en
